# Better Quality Sleep Promotes Daytime Physical Activity in Patients with Chronic Pain? A Multilevel Analysis of the Within-Person Relationship

**DOI:** 10.1371/journal.pone.0092158

**Published:** 2014-03-25

**Authors:** Nicole K. Y. Tang, Adam N. Sanborn

**Affiliations:** Department of Psychology, University of Warwick, Coventry, United Kingdom; University of Louisville, United States of America

## Abstract

**Background:**

Promoting physical activity is key to the management of chronic pain, but little is understood about the factors facilitating an individual’s engagement in physical activity on a day-to-day basis. This study examined the within-person effect of sleep on next day physical activity in patients with chronic pain and insomnia.

**Methods:**

119 chronic pain patients monitored their sleep and physical activity for a week in their usual sleeping and living environment. Physical activity was measured using actigraphy to provide a mean activity score each hour. Sleep was estimated with actigraphy and an electronic diary, providing an objective and subjective index of sleep efficiency (A-SE, SE) and a sleep quality rating (SQ). The individual and relative roles of these sleep parameters, as well as morning ratings of pain and mood, in predicting subsequent physical activity were examined in multilevel models that took into account variations in relationships at the ‘Day’ and ‘Participant’ levels.

**Results:**

Of the 5 plausible predictors SQ was the only significant within-person predictor of subsequent physical activity, such that nights of higher sleep quality were followed by days of more physical activity, from noon to 11pm. The temporal association was not explained by potential confounders such as morning pain, mood or effects of the circadian rhythm.

**Conclusions:**

In the absence of interventions, chronic pain patients spontaneously engaged in more physical activity following a better night of sleep. Improving nighttime sleep may well be a novel avenue for promoting daytime physical activity in patients with chronic pain.

## Introduction

As the fourth leading risk factor for noncommunicable diseases, physical inactivity is now considered a global pandemic with approximately 31% of adults worldwide reporting a pattern of physical activity that falls short of the World Health Organization recommendations [1], [2]. Although there is little ambiguity about the need to promote physical activity, it remains elusive what constitutes an effective method to increase physical activity. Common ways to promote physical activity in community health care settings include verbal advice, referral to an exercise programme, the use of a pedometer, and enrollment in a walking and/or cycling scheme [3]–[5]. However, the evidence base for the long-term effectiveness of these strategies is limited and the factors determining an individual’s capability to engage in physical activity on a day-to-day basis are yet to be identified.

It is a particular challenge promoting physical activity in people suffering from chronic pain, which is pain that persists beyond the normal expected time for healing (1–6 months) [6]. Whilst some people manage to live well despite pain, many experience elevated levels of distress and disability as pain has the natural physical and emotional qualities to interrupt activities [7], [8]. Although activity interruptions serve to protect our physical integrity when pain is acute, prolonged disengagement from activities may result in physical deconditioning, economic loss and further emotional distress as a result of the loss of psychosocial functions [9]–[11]. Promoting physical activity is therefore a key treatment goal in the management of chronic pain.

Contemporary psychological theories of chronic pain have highlighted the role of pain catastrophising and habitual coping strategies in determining a person’s engagement in physical activity. Across a number of fear-avoidance models [12]–[18], it has been suggested that individuals with greater fear of pain, physical movement or reinjury are more likely to display activity avoidance and a lower level of physical activity compared to those who are less fear-avoidant. It has also been suggested that highly fluctuating levels of physical activity may be observed in a subgroup of pain patients who have a tendency to persevere through tasks until pain is unbearable [19], [20]. Although these accounts are compelling, a handful of recent studies that examined the association of fear-avoidance or pain-endurance on daily physical activity did not find evidence in support of these theories.

Huijnen et al. [21] classified 79 chronic low back pain patients into “avoiders”, “persisters”, and “mixed performers”. Whilst these patients all reported higher levels of disability and lower levels of physical activity compared to “functional performers”, no between-group differences were observed in their daily physical activity objectively measured with an accelerometer over 14 consecutive days. The correlation between pain and daily physical activity was non-significant. Similarly, Helmus et al. [22] also reported non-significant correlations between habitual coping strategies (active or passive coping, activity avoidance) and objectively assessed physical activity in their cross-sectional study involving 53 patients with chronic musculoskeletal pain. Using a longitudinal design, Leonhardt et al. [23] examined the influence of fear avoidance beliefs on the levels of physical activity reported one year later in 787 patients with acute and chronic lower back pain. Structural equation analysis revealed that fear avoidance beliefs were non-significant predictors of physical activity at 1-year, which remained largely the same throughout the year. These findings converge to suggest that the between-person difference in physical activity by fear-avoidance beliefs or habitual coping strategies is possibly negligible, and that in people with chronic pain, pain intensity is unlikely the primary predictor of their day-to-day physical activity. For the development of novel strategies for promoting physical activity in patients with chronic pain, it may be more fruitful to examine the within-person factors that explain variations in physical activity across times.

One possible within-person factor involved in the regulation of daily physical activity in chronic pain is sleep, a behavioural state characterised by a relative absence of physical activity. Sitting on different ends of the same continuum, the oscillation between sleep and physical activity is a key dimension defining a person’s sleep-wake cycle. It has been proposed that sleep disturbance interacts with central pain processing and inflammatory mechanisms to augment pain, low mood and poorer physical functioning [24]. Whilst there is growing evidence to indicate a negative effect of sleep disruption on pain and mood reports [25]–[31], the impact of sleep disturbance on pain patients’ subsequent physical activity is only beginning to be investigated. There is initial evidence suggesting that, among young adults with parental history of type 2 diabetes, those with shorter sleep duration (<6 hr per night) engaged in less physical activity than their counterparts with longer sleep duration (≥6 hr per night) [32]. Conversely, some correlational evidence drawn from older adults suggests that sleep of better quality is associated with higher walking speed, faster completion of sit-to-stand tasks, and less self-reported limitations on activities of daily living [33], [34]. Whilst none of these studies demonstrates a direct effect of sleep on subsequent physical activity, their findings highlight the possibility of increasing chronic pain patients’ spontaneous engagement in physical activity through improving sleep.

The current study examined the role of sleep in the regulation of physical activity among chronic pain patients with concomitant insomnia. Specifically, we aimed to determine whether day-to-day fluctuations in sleep have an impact on patients’ physical activity the following day. A daily process approach was used focussing on the within-person relationship of sleep with physical activity in chronic pain individuals [35]. This approach allowed us to ascertain the presence/absence of a temporal relationship within an individual and to gauge the broader benefits of sleep interventions for chronic pain patients. Physical activity was measured using actigraphy to provide an objective estimate of physical activity around the clock. It was hypothesised that if sleep serves a recuperative function for chronic pain patients, a night of better-quality sleep would be followed by a higher level of physical activity the next day.

## Materials and Methods

### Ethics Statement

The protocol of the research received full ethical approval from the Institute of Psychiatry/South London and Maudsley NHS Research Ethics Committee (Ref: 06/Q0706/125). All participants provided a written informed consent before taking part in the study.

### Overview

**Figure 1:**
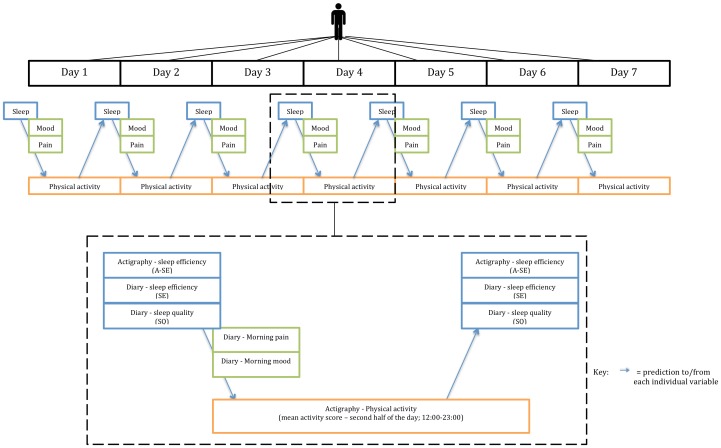
Design and analysis plan of the study.

We analysed data collected in a recent daily process study involving 119 patients presenting with chronic pain and insomnia. The protocol of the study was described in full elsewhere [36]. Briefly, all participants were asked to monitor their sleep and physical activity by wearing an actigraph round-the-clock for a week. In addition, they were asked to keep an electronic diary to provide subjective estimates of their sleep quality and sleep efficiency, as well as ratings of pain and mood at different times of the day throughout the study. Applying multilevel modeling on the time-specific data, we assessed the impact of within-person changes in sleep quality and efficiency on physical activity levels during the following day. Although previous studies found neither pain nor mood a significant predictor of next-day physical activity [37], [38], we were mindful of the influence of sleep on these variables and included participants’ morning ratings of pain and mood in our models to control for these potential confounding factors and to maximise comparability of the current findings with the literature. Figure 1 depicts the design and data analysis plan of the current study.

### Participants

Participants were patients recruited consecutively from a hospital pain clinic in London, UK. Inclusion criteria were: working-age adults between 18 and 65 years; English-speaking; non-malignant pain of at least 6 months; scoring 15 or higher on the Insomnia Severity Index ([39]; indicating clinical insomnia). Exclusion criteria were: recent (i.e., past month) or impending (i.e., during the duration of the study) surgical procedure for pain reduction; medical conditions indicative of pain of malignant nature (e.g., cancer, HIV/AIDS); severe psychiatric or psychological problems with acute distress (e.g., psychosis, major depression with suicide intent); visual or cognitive impairments that interfered with the monitoring and assessment procedure (e.g., poor vision, dementia).

**Figure 2:**
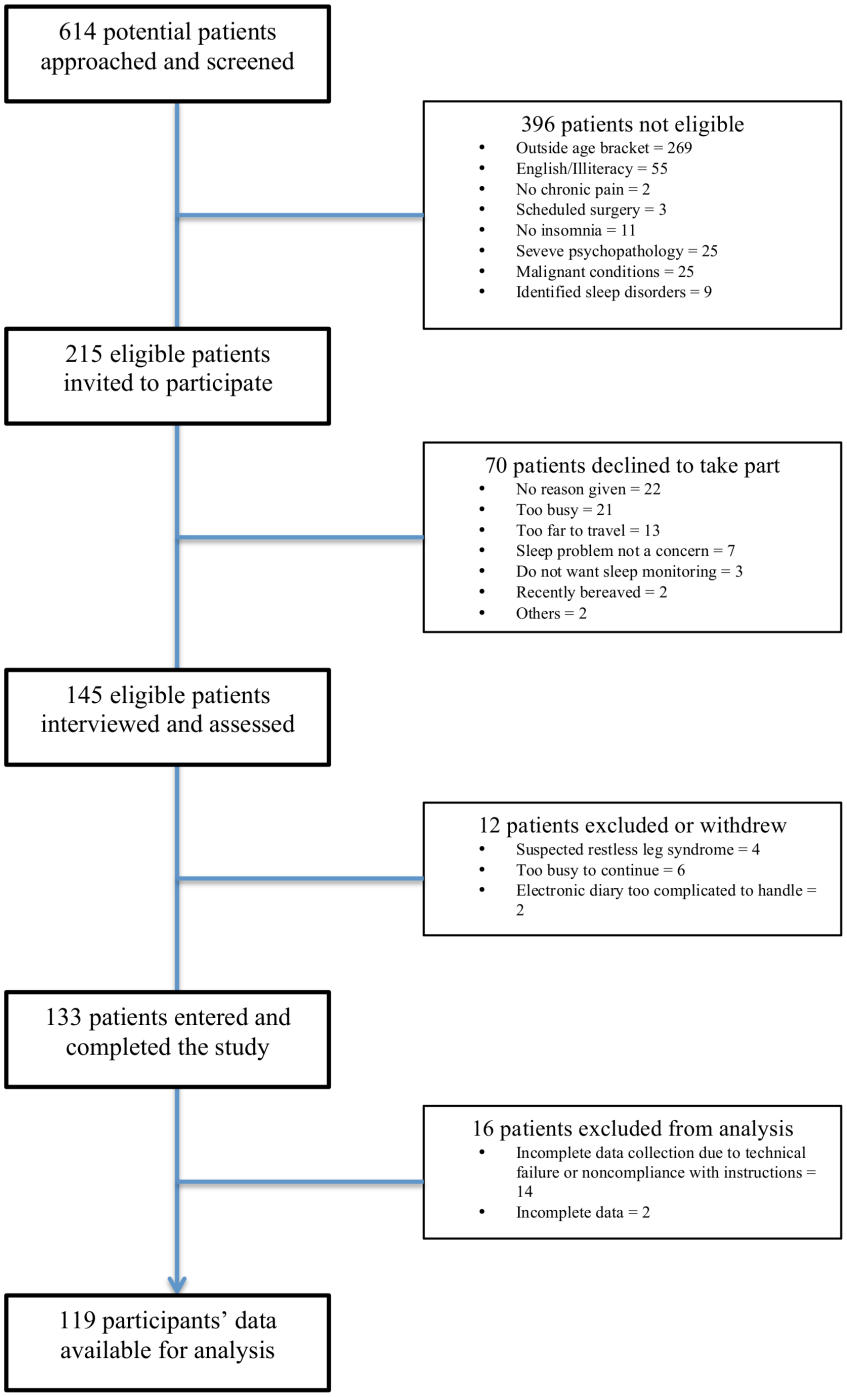
Participant recruitment flowchart.

Participants’ eligibility was assessed by an experienced health psychologist using a checklist of inclusion and exclusion criteria. In addition, the Duke Structured Interview Schedule for DSM-IV-TR and ICSD-2 [40] was administered to confirm the presence of insomnia complaints that met the American Academy of Sleep Medicine research diagnostic criteria [41] and that, aside from pain, there were no other medical, psychiatric, or sleep disorders that could better account for the insomnia. For the current study, complete data from a total of 119 patients were available for analysis (see Figure 2 for a recruitment flow diagram). The majority of the participants had more than one pain location (87%). Lower back (73%) was the commonest site of pain, followed by legs (54%), neck (38%), shoulders (33%), knees (35%), arms (21%), upper back (22%) and joints (22%).

### Materials

#### Actigraphy

Actigraphy was used to provide an objective estimate of sleep during the night and to index the level of physical activity during the day. It is a lightweight, nonintrusive device to be worn on the nondominant wrist, similar to a normal wrist watch. The device contains a piezoelectric accelerometer set up to record the integration, amount, and duration of movements. The corresponding voltage (Hz) is then converted and stored as activity count data, which are then downloaded for activity and sleep analysis using the software, Actiwatch Activity and Sleep Analysis (supplied by Cambridge Neurotechnology Ltd., Cambridge, UK) version 5.43.

The Actiwatch-Insomnia model was used in an attempt to improve specificity in detecting quiet wakefulness. A small pressure sensor, which is not a device used with conventional actigraphs, is attached to the watch to be held by the wearer between the thumb and the finger until muscle tone relaxes at the onset of sleep. This additional behavioural measure of sleep onset facilitates the scoring of sleep onset latency and has been shown to improve accuracy in the estimation of wakefulness [42], and thus the calculation of the sleep efficiency – a widely recognised index of sleep consolidation/fragmentation. As per standard protocol, the epoch length was set to 0.5 min. The participants were asked to depress the event marker once when they switched off the light and got ready for bed and once when they got up in the morning. To facilitate the scoring and detection of awakenings, the participants were also asked to hold the pressure sensor with their fingers as they tried to fall asleep and every time when they woke up from sleep. The validity of using actigraphy to characterise and monitor sleep patterns and circadian rhythms has been confirmed by the Standards of Practice Committee of the American Academy of Sleep Medicine based on a systematic grading of evidence by a panel of content experts with expertise in the use of the technology [43].

In the current study, key variables derived from the actigraphic data for analyses were: (i) Actigraphic sleep efficiency index (A-SE), which has been found a valid measure of sleep pattern in community volunteers with comorbid insomnia [44], and (ii) Mean activity score by hour. Activity values below 10 were coded as missing observations.

#### Electronic Diary

The electronic daily diary was custom-built for the current study using Satellite Forms version 7.2 (supplied by Thacker Network Technologies Inc., Canada). It was operated on handheld computers (PalmPDA, model: Z22, Palm, Inc., Sunnyvale CA) that had a touch-screen interface, allowing the participants to enter their response using a stylus pen. Each completed diary was time-stamped, locked and saved in the handheld computer, preventing late and retrospective data entries. Diaries not completed before the next diary was due were considered “expired”. Expired diaries were also automatically locked and saved to safeguard the timeliness of the data collected. It has been shown in previous research that compared to paper diaries, electronic diaries enhance chronic pain patients’ compliance to the monitoring procedure to above 90% [45].

Subjective sleep estimates provided by the participants everyday on waking included: sleep onset latency (SOL; how long it had taken them to fall asleep), wake after sleep onset (WASO; times woken up after sleep onset), duration of wake after sleep onset (WASO duration; how long they had been woken up after sleep onset), total sleep time (TST; how long they had slept all together), and sleep quality (SQ; “How would you rate the quality of sleep obtained last night?”; 0–10 numeric rating scale (NRS): 0  =  “very poor”, 10  =  “very good”).

The use of the daily diary methodology, electronic or paper-based, is widely applied to the study of sleep and pain [46], [47]. The methodology allowed us to sample experience or events as they happened. It provided dynamic data on within-person change over time that could not be obtained from cross-sectional surveys or objective tests with an infrequent assessment schedule [48]. Mixed findings have been published regarding participants’ reactance, habituation and gradual entrainment as a result of the act of repeated measurement. The increase in awareness of the monitored behaviour did not consistently result in reactance in the behaviour itself [36], [49], [50]. In cases where reactivity was reported, the effect tended to dissipate within two to three days [48].

The key sleep-diary variables used in the current analysis were: (i) Sleep Quality, (ii) Sleep Efficiency, as calculated by: [TST/ (SOL +WASO duration +TST)] x 100%, as well as (iii) subjective ratings of pain (“How much pain do you have right now?”; 0–10 NRS; 0  =  ”no pain at all”, 10  =  ”a lot of pain”) and mood (“How would you describe your mood right now?”; 0–10 NRS; 0  =  “very bad mood”, 10 =  “very good mood”) provided by the participants everyday on waking.

### Procedure

Ambulatory monitoring was used to maximise the ecological validity of the study. In their usual sleeping and living environment, participants were asked to monitor their sleep, pain, mood and activity using the equipment described above for a week. All but 2 of the 119 participants completed 7 days of monitoring; 118 completed 6 days and all 119 completed 5 days of monitoring.

Written informed consent was obtained from each participant at the start of the study, when they attended a training session in which the researcher explained the rationale and procedure of the research. Specifically, they were told that the study aimed to examine their typical sleep-wake pattern and they were explicitly instructed to not change their usual activity pattern, sleeping and working environment, use of medication and substances (e.g., alcohol, tobacco, caffeine) throughout the duration of the study. Moreover, to enhance compliance and accuracy of data collection, each participant was given individual training on using the actigraph and the handheld computer that displayed the electronic diary. They were instructed to wear the actigraph on their non-dominant wrist day and night except when coming into contact with water. They were shown how to enter data in the electronic diary and navigate between pages, and urged to complete the diary as soon as prompted by the alarm, which was set to go off three times a day according to their typical bedtime and rise time. Whilst three diaries were to be completed daily by each participant on waking (diary 1), just before bed (diary 3) and at the midpoint between diaries 1 and 3 (diary 2), only diary 1 contained subjective sleep estimates and other data relevant to the current analysis. The participants were loaned the equipment to carry out the monitoring task once they had shown understanding of the full procedure and completed a full set of training diaries. They were also given a handbook with step-by-step photographic instruction to take home as a reference, and encouraged to contact the investigator as soon as convenient should any problem arise. The participants returned a week later with the equipment to have the data downloaded. They were asked to report any unexpected changes to their typical sleep-wake schedule and any technical issues with the actigraph and the handheld computer. After debriefing, each participant received a £20 gift voucher as an honorarium.

### Data analysis

To evaluate the within-person temporal link between sleep and physical activity the following day, we pooled together the daily monitoring data from all participants, generating an aggregate data set of 830 observations. The individual and relative role of the three key sleep parameters (sleep quality, sleep efficiency and actigraphy sleep efficiency) and morning pain and mood ratings in predicting subsequent physical activity levels were examined. Any observations in which one or more of the variables of interest were missing were removed, yielding 754 observations for analysis. The statistical language R with the “lme4” package was used to carry out multilevel analysis on the observations, taking into account variations in the relationship between sleep and activity at both the ‘Day’ level (Level 1) and the ‘Participant’ level (Level 2). We performed a between-model comparison to enable us to both determine whether predictors were significant as well as determine the relative strength of the various predictors of interest.

We first fit multilevel models to examine which aspects of sleep predicted the mean activity score over the second half of the day (from noon to 11pm). The morning diary data used as predictors were taken before noon, so using this range of hours improved the specificity of the temporal prediction by giving a clearer chronological order of the events. Using the mean activity score over the entire day as the dependent variable resulted in the same ordering of the relative strengths of the predictors. We compared the role of sleep quality (SQ), sleep efficiency (SE), actigraphy sleep efficiency (A-SE), mood upon waking (Morning Mood) and pain upon waking (Morning Pain) in predicting subsequent physical activity. In each set of the analysis, the first model was always the one that only included a constant fixed term.

In the results section below, we assessed the significance of each predictor by comparing it to a constant-only model (i.e., the baseline model that lacked the predictor) using a Likelihood Ratio Test. In addition, we directly compared the strengths of the predictors to each other using Akiake Information Criterion (AIC) values, which trade off goodness of fit against a penalty for model complexity. Smaller AIC values indicate better models, and the differences between AIC values indicate the relative strength of predictors. These differences were then assessed in the form of probabilities, where larger values are better. Details of this method are given in Appendix S1. In the results tables, we also report the fixed coefficients for the best models (in terms of AIC values) to indicate the direction of the relationship.

## Results

### Participant characteristics

Participants included in the current analysis had a mean age of 46 (SD = 10.9) and a mean body mass index (BMI) of 27.7 (SD = 6.1). The majority of them are Caucasian (76%) and female (74%). Just under half of them (48%) were married or living as married, and the same percentage of participants were on sick leave/unemployed at the time of the study (48%). As a group, the participants reported a mean pain and insomnia duration of 10.4 (SD = 9.6; Median = 8) and 7.9 years (SE = 8.3; Median = 5), respectively. Their mean ISI score (20.1) was well above the cut-off for clinical insomnia [39].

### Predicting mean physical activity score over the second half of the day

Models of the effect of sleep, mood, and pain (i.e., SQ, A-SE, SE, Morning Mood, Morning Pain) on mean physical activity score over the second half of the day (noon to 11pm) were compared. Table 1 gives the model components, fixed coefficients of the predictor(s) the negative log maximum likelihood values (larger is better), number of parameters, the significance of the predictor(s), the AIC value which corrects for the number of parameters (smaller is better), and the relative probability of each model (larger is better) as determined from the AIC values.

**Table 1:** A summary of model outcomes in predicting mean physical activity during the second half of the day (noon to 11pm).

Model Terms	Fixed Coefficients	Tests for Model Selection			
		LRT		AIC	
		-Log Likelihood	Significance	Value	Relative Probability
Constant (C)	307	4598	n/a	9207	0.11
SQ + C	290, 4.07	4597	p = .017	9204	0.57
A-SE + C	357, –0.528	4597	p = .474	9209	0.05
SE + C	280, 35.7	4599	p = .190	9208	0.08
Morning Mood + C	290, 4.08	4598	p = .079	9206	0.16
Morning Pain + C	314, –1.13	4600	p = .581	9209	0.04

SQ  =  Sleep quality; A-SE  =  actigraphy sleep efficiency; SE  =  diary sleep efficiency; n/a  =  not applicable.

LRT  =  Likelihood Ratio Test, which assessed the significance of each predictor of interest (e.g., SQ) by comparing the alternative model (e.g., SQ + C; –Log Likelihood  =  4597) with the appropriate null model with only a constant term. The p value adjacent indicated whether the alternative model was significantly better.

AIC  =  Akiake Information Criterion, unlike LRT, compared the alternative models directly (e.g., SQ + C versus A-SE + C), taking into account each model’s complexity. Smaller AIC values indicate better models, but the absolute sizes of the AIC values are not informative. Instead the difference between them indicates the relative strength of predictors, which were then assessed in the form of relative probabilities, where larger values are better.

As can be seen from Table 1, A-SE, SE, and Morning Pain were not significant predictors of mean physical activity score over the second half of the day (all *p* values >.1). SQ was a significant predictor of physical activity (*p* = .017), and Morning Mood was near the significance threshold (*p*  = .079). Amongst all predictors considered in this set of analysis, SQ was the best predictor of mean physical activity score over the second half of the day, with a relative probability of .57.

**Figure 3:**
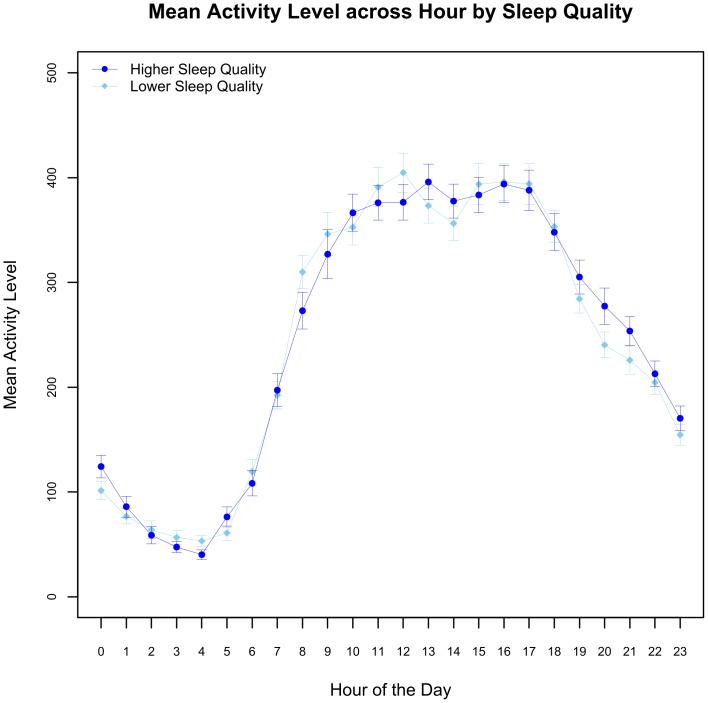
A comparison of mean physical activity level by hour of the day between days following nights of highest individual sleep quality and those following nights of lowest individual sleep quality. There was a clear circadian rhythm of physical activity overall, but higher levels of physical activity were seen in participants who had had a night of better quality sleep.

A more fine-grained view of the effect of SQ on physical activity is shown in Figure 3, which depicts the participants’ 24-hour pattern of physical activity following the highest rated nights of sleep quality and the lowest rated nights of sleep quality. This plot shows a clear circadian pattern for both types of days, with a visual trend of increasing physical activity between 4am and 10am, a high level of activity being maintained between 10am and 4pm, and a gradual decline in physical activity from 4pm till 4am. The pattern of peak and trough was different between the highest and lowest sleep quality days; between 10am and 4pm, individuals had a more fluctuating activity on the lowest sleep quality days and a more prominent ‘post-lunch dip’. However, the magnitude of the difference was small in comparison to the intra-daily variation.

## Discussion

The level of physical activity varies both between people and within an individual across different times and days. Factors distinguishing the physically active from the physically inactive group may not be the same as those that alter a person’s capacity to engage in physical activity on a day-to-day basis [35]. Motivated by a recent theory describing how sleep disturbance interacts with central pain and inflammatory processes to augment pain, low mood and poorer physical functioning [24], the current study was the first to investigate sleep as a possible within-person factor that determines the level of physical activity the next day.

Multilevel modeling was applied to analyse the temporal patterns in 830 sets of data drawn from 119 chronic pain patients who kept a record of their sleep and physical activity for a week. The findings indicated that, despite the presence of chronic pain, nights of higher sleep quality were followed by days of higher levels of physical activity. This association with sleep quality was observed for the mean level of physical activity during the second half of the day (noon to 11pm). Although a causal relationship cannot be inferred, this finding provided a good illustration of the sequential association as it incorporated a clear chronological order of the predictor and the predicted variable, minimising the risk of inflating the strength of the sleep-physical activity relationship due to overlaps in measurements. It also supported the recuperation hypothesis that better sleep enhances chronic pain patients’ capability to engage in physical activity.

However, not all sleep parameters were significant predictors of subsequent physical activity; sleep efficiency indices respectively calculated using sleep diary and actigraphy data were not significant predictors of physical activity the following day. This is surprising considering that SE is commonly used as an indicator of sleep consolidation and that it has been found to be correlated with SQ in previous research [51], [52] and in the current study (r = 0.46). This pattern of findings underscore the qualitative difference between the two sleep parameters, and it seems plausible that a person’s subjective perception of their sleep quality carries a stronger influence on subsequent physical activity than their objective sleep experience. In addition to replicating the findings, it would be important for future research to investigate the pathways through which the perception of good quality sleep increases physical activity. Our previous work indicated that chronic pain patients reported less pain in the morning following a night of better quality sleep [36]. However, in the current study morning pain was not a significant within-person predictor of subsequent physical activity and so it seems unlikely that pain is a mediator of the sleep-physical activity relationship. The same argument applies to morning mood, which was not found to be a significant within-person predictor of subsequent physical activity. These findings were in agreement with the specifics of the Smith et al. [24] model that sleep disruption may interact with multiple mechanisms other than pain and mood to impact on physical function. As the next step, experimental research incorporating quantitative sensory testing and measurements of inflammation and the neuroendocrine functioning will help illuminate the biological pathways through which sleep impacts on physical activity regulation. Qualitative studies examining pain patients’ spontaneous and meditated reactions to perceived good quality sleep may also shed light on the psychosocial pathways through which better SQ motivates subsequent engagement in physical activity. Moreover, Harvey and colleagues [53] showed that compared with normal sleepers, patients with insomnia tend to have more requirements for judging their sleep quality, defining their sleep quality not only by their sleep experience and how they feel immediately on waking but also by tiredness detected on waking and during the day. It might be fruitful for future research to investigate the effect of tiredness or fatigue on sleep perception and identify other criteria chronic pain patients use to assess their day-to-day sleep quality.

Methodologically, the prospective design of the current study is a strength. Through the use of time-lagged data analysis, we could establish temporal precedence of the sleep-physical activity association. Repeated measurements were taken for sleep and physical activity from each participant, and their data collected on different days were pooled together to generate a larger data set to increase the power of the analysis. The potential issue of reactivity should be noted [54], [55]. Although previous studies have shown that the procedure of electronic diary assessment was nonreactive [36], [49], a post hoc analysis indicated that, when ‘Day’ was included as a lone factor in a model, it was a significant predictor of SE and mean physical activity in the second half of the day but not for SQ, A-SE, Morning mood or Morning Pain. The direction of the reactivity effects showed a decrease in physical activity and an increase in SE over days. The trends combined appeared to suggest a gradual habituation process as the participants relaxed into the monitoring procedure. Indeed, a visual inspection of the data indicated that the decline in mean physical activity and the increase in SE levelled off after Day 3. Future research using the daily process design should consider lengthening the sampling time frame and allowing at least 3 days for adaptation purposes, although this will inevitably increase the research cost and the burden on participants.

Objective estimates of sleep and physical activity were provided by uniaxial actigraphy. Whilst actigraphy has the advantage of being light-weight, non-intrusive, and cost-effective, it does not provide information about sleep staging, architecture, and spectral abnormality. The activity count data generated do not inform the type and content of the physical activities involved. This makes it impossible to judge whether the increase in physical activity counts translates into any clinical meaningful improvement to the patients. Moreover, the use of wrist-worn uniaxial accelerometers may underestimate physical activities that do not involve wrist or arm movement. New generations of triaxial accelerometers should be able to provide more precise information for the calculation of energy expenditure.

Finally, the prospect of promoting physical activity by regulating sleep may offer a novel solution to an old problem. We focused on patients with chronic pain because sleep disturbance and reduced physical activity are common consequences of this clinical population. Further research should establish whether the current findings generalise to other long term conditions that are characterised by sleep disturbance and reduced physical activity to varying degree (e.g., asthma, chronic obstructive pulmonary disorders, diabetes, fibromyalgia, high blood pressure, and obesity).

Despite the limitations discussed above, the current study identified sleep quality rather than pain and low mood as a key driver of physical activity the next day. In the absence of any intervention, chronic pain patients having had a better night of sleep spontaneously engaged in more physical activity the following day. This suggests a naturally energising function of sleep and highlights the often-overlooked continuity between nighttime sleep and daytime physical activity. Existing strategies for promoting physical activity tend to focus on actions during the day. Additional efforts in promoting sleep among physically inactive subgroups may increase the overall impact of these interventions.

## Supporting Information

Appendix S1 **Details of the analysis method.**
(DOCX)

## References

[1] Hallal PC, Andersen LB, Bull FC, Guthold R, Haskell W, et al (2012) Global physical activity levels: surveillance progress, pitfalls, and prospects. The Lancet 380: 247–257. 10.1016/S0140-6736(12)60646-1 22818937

[2] World Health Organization (2010) Global recommendations on physical activity for health. Switzerland: WHO Press. 26180873

[3] National Institute for Clinical Excellence (2006) PH2 Four commonly used methods to increase physical activity: guidance.

[4] National Institute for Clinical Excellence (2013) PH44 Physical activity: brief advice for adults in primary care: guidance.

[5] National Institute for Clinical Excellence (2012) PH41 Walking and cycling: guidance.

[6] The IASP Taxonomy Working Group (2013) Classification of Chronic Pain. Descriptions of Chronic Pain Syndromes and Definitions of Pain Terms. Second Edition (Revised). Washington, DC: IASP Press.

[7] Eccleston C, Crombez G (1999) Pain demands attention: A cognitive–affective model of the interruptive function of pain. Psychological bulletin 125: 356. 10349356 10.1037/0033-2909.125.3.356

[8] Van Damme S, Legrain V, Vogt J, Crombez G (2010) Keeping pain in mind: a motivational account of attention to pain. Neuroscience & Biobehavioral Reviews 34: 204–213. 19896002 10.1016/j.neubiorev.2009.01.005

[9] Harris S, Morley S, Barton SB (2003) Role loss and emotional adjustment in chronic pain. Pain 105: 363–370. 14499455 10.1016/s0304-3959(03)00251-3

[10] Verbunt J, Seelen H, Vlaeyen JWS (2004) Disuse and physical deconditioning in chronic low back pain. In: Asmundson GJG, Vlaeyen JWS, Crombez G, editors. Understanding and Treating Fear of Pain. New York: Oxford University Press. pp. 139–160.

[11] Pincus T, Morley S (2001) Cognitive-processing bias in chronic pain: a review and integration. Psychological Bulletin 127: 599–617. 11548969 10.1037/0033-2909.127.5.599

[12] Asmundson GJG, Norton GR, Allerdings MD, Norton PJ, Larsen DK (1998) Post traumatic stress disorder and work-related injury. J Anxiety Disord 12: 57–69. 9549609 10.1016/s0887-6185(97)00049-2

[13] Lethem J, Slade P, Troup J, Bentley G (1983) Outline of a fear-avoidance model of exaggerated pain perception—I. Behaviour research and therapy 21: 401–408. 6626110 10.1016/0005-7967(83)90009-8

[14] Asmundson GJ, Norton PJ, Norton GR (1999) Beyond pain: the role of fear and avoidance in chronicity. Clinical psychology review 19: 97–119. 9987586 10.1016/s0272-7358(98)00034-8

[15] Philips H (1987) Avoidance behaviour and its role in sustaining chronic pain. Behaviour research and therapy 25: 273–279. 3662989 10.1016/0005-7967(87)90005-2

[16] Vlaeyen JW, Kole-Snijders AM, Boeren RG, van EH (1995) Fear of movement/(re)injury in chronic low back pain and its relation to behavioral performance. Pain 62: 363–372. 8657437 10.1016/0304-3959(94)00279-N

[17] Vlaeyen JW, Linton SJ (2000) Fear-avoidance and its consequences in chronic musculoskeletal pain: A state of the art. Pain 85: 317–332. 10781906 10.1016/S0304-3959(99)00242-0

[18] Waddell G, Newton M, Henderson I, Somerville D, Main CJ (1993) A Fear-Avoidance Beliefs Questionnaire (FABQ) and the role of fear-avoidance beliefs in chronic low back pain and disability. Pain 52: 157–168. 8455963 10.1016/0304-3959(93)90127-B

[19] Hasenbring M, Marienfeld G, Kuhlendahl D, Soyka D (1994) Risk factors of chronicity in lumbar disc patients: a prospective investigation of biologic, psychologic, and social predictors of therapy outcome. Spine 19: 2759–2765. 7899975 10.1097/00007632-199412150-00004

[20] Hasenbring MI, Verbunt JA (2010) Fear-avoidance and endurance-related responses to pain: new models of behavior and their consequences for clinical practice. The Clinical journal of pain 26: 747–753. 20664333 10.1097/AJP.0b013e3181e104f2

[21] Huijnen IPJ, Verbunt Ja, Peters ML, Smeets RJEM, Kindermans HPJ, et al (2011) Differences in activity-related behaviour among patients with chronic low back pain. European Journal of Pain 15: 748–755. 21195646 10.1016/j.ejpain.2010.11.015

[22] Helmus M, Schiphorst Preuper H, Hof A, Geertzen J, Reneman M (2012) Psychological factors unrelated to activity level in patients with chronic musculoskeletal pain. European Journal of Pain 16: 1158–1165. 22337000 10.1002/j.1532-2149.2011.00109.x

[23] Leonhardt C, Lehr D, Chenot J-F, Keller S, Luckmann J, et al.. (2009) Are fear-avoidance beliefs in low back pain patients a risk factor for low physical activity or vice versa? A cross-lagged panel analysis. GMS Psycho-Social-Medicine 6. 10.3205/psm000057 PMC2736477 19742047

[24] Smith M, Quartana P, Okonkwo R, Nasir A (2009) Mechanisms by which sleep disturbance contributes to osteoarthritis pain: a conceptual model. Current Pain and Headache Reports 13: 447–454. 19889286 10.1007/s11916-009-0073-2

[25] Haack M, Mullington JM (2005) Sustained sleep restriction reduces emotional and physical well-being. Pain 119: 56–64. 16297554 10.1016/j.pain.2005.09.011

[26] Lentz MJ, Landis CA, Rothermel J, Shaver JLK (1999) Effects of selective slow wave sleep disruption on musculoskeletal pain and fatigue in middle aged women. Journal of Rheumatology 26: 1586–1592. 10405949

[27] Moldofsky H, Scarisbrick P (1976) Induction of neurasthenic musculoskeletal pain syndrome by selective sleep stage deprivation. Psychosomatic Medicine 38: 35–44. 176677 10.1097/00006842-197601000-00006

[28] Morin CM, Gibson D, Wade J (1998) Self-reported sleep and mood disturbance in chronic pain patients. Clinical Journal of Pain 14: 311–314. 9874009 10.1097/00002508-199812000-00007

[29] Roehrs T, Hyde M, Blaisdell B, Greenwald M, Roth T (2006) Sleep loss and REM sleep loss are hyperalgesic. Sleep 29: 145–151. 16494081 10.1093/sleep/29.2.145

[30] Smith MT, Edwards RR, McCann UD, Haythornthwaite JA (2007) The effects of sleep deprivation on pain inhibition and spontaneous pain in women. Sleep 30: 494–505. 17520794 10.1093/sleep/30.4.494

[31] Tang NKY, Wright KJ, Salkovskis PM (2007) Prevalence and correlates of clinical insomnia co-occurring with chronic back pain. Journal of Sleep Research 16: 85–95. 17309767 10.1111/j.1365-2869.2007.00571.x

[32] Booth JN, Bromley LE, Darukhanavala AP, Whitmore HR, Imperial JG, et al (2012) Reduced physical activity in adults at risk for type 2 diabetes who curtail their sleep. Obesity 20: 278–284. 21996665 10.1038/oby.2011.306 PMC3262101

[33] Dam TTL, Ewing S, Ancoli-Israel S, Ensrud K, Redline S, et al (2008) Association between sleep and physical function in older men: the osteoporotic fractures in men sleep study. Journal of the American Geriatrics Society 56: 1665–1673. 18759758 10.1111/j.1532-5415.2008.01846.x PMC2631084

[34] Goldman SE, Stone KL, Ancoli-Israel S, Blackwell T, Ewing SK, et al (2007) Poor sleep is associated with poorer physical performance and greater functional limitations in older women. Sleep 30: 1317. 17969465 10.1093/sleep/30.10.1317 PMC2266278

[35] Affleck G, Zautra AJ, Tennen H, Armeli S (1999) Multilevel daily process designs for consulting and clinical psychology: a preface for the perplexed. Journal of Consulting and Clinical Psychology 67: 746–754. 10535241 10.1037//0022-006x.67.5.746

[36] Tang NKY, Goodchild CE, Sanborn AN, Howard J, Salkovskis PM (2012) Deciphering the temporal link between pain and sleep in a heterogeneous chronic pain patient sample: A multilevel daily process study. Sleep 35: 675–687. 22547894 10.5665/sleep.1830 PMC3321427

[37] Huijnen IPJ, Verbunt Ja, Peters ML, Delespaul P, Kindermans HPJ, et al (2010) Do depression and pain intensity interfere with physical activity in daily life in patients with Chronic Low Back Pain? Pain 150: 161–166. 20457489 10.1016/j.pain.2010.04.021

[38] Vendrig aa, Lousberg R (1997) Within-person relationships among pain intensity, mood and physical activity in chronic pain: a naturalistic approach. Pain 73: 71–76. 9414058 10.1016/s0304-3959(97)00075-4

[39] Bastien C, Valliäres A, Morin CM (2001) Validation of the Insomnia Severity Index as a clinical outcome measure for insomnia research. Sleep Medicine 2: 297–307. 11438246 10.1016/s1389-9457(00)00065-4

[40] Edinger JD, Kirby AC, Lineberger MD, Loiselle MM, Wohlgemuth WK, et al.. (2006) DUKE structured interview schedule for DSM-IV-TR and International Classification of Sleep Disorders, second edition (ICSD-2) sleep disorder diagnoses. Durham, North Carolina: Veterans Affairs and Duke University Medical Centers.

[41] Edinger JD, Bonnet MH, Bootzin RR, Doghramji K, Dorsey CM, et al (2004) Derivation of research diagnostic criteria for insomnia: report of an American Academy of Sleep Medicine Work Group. Sleep 27: 1567–1596. 15683149 10.1093/sleep/27.8.1567

[42] Pilsworth S, King M, Shneerson J, Smith I (2001) A comparison between measurements of sleep efficiency and sleep latency measured by polysomnography and wrist actigraphy. Sleep 24: A106.

[43] Morgenthaler T, Alessi C, Friedman L, Owens J, Kapur V, et al (2007) Practice parameters for the use of actigraphy in the assessment of sleep and sleep disorders: an update for 2007. Sleep 30: 519. 17520797 10.1093/sleep/30.4.519

[44] Lichstein KL, Stone KC, Donaldson J, Nau SD, Soeffing JP, et al (2006) Actigraphy validation with insomnia. SLEEP-NEW YORK THEN WESTCHESTER- 29: 232. 16494091

[45] Stone AA, Shiffman S, Schwartz JE, Broderick JE, Hufford MR (2003) Patient compliance with paper and electronic diaries. Controlled Clinical Trials 24: 182–199. 12689739 10.1016/s0197-2456(02)00320-3

[46] Carney CE, Buysse DJ, Ancoli-Israel S, Edinger JD, Krystal AD, et al (2012) The consensus sleep diary: standardizing prospective sleep self-monitoring. Sleep 35: 287. 22294820 10.5665/sleep.1642 PMC3250369

[47] Haythornthwaite JA, Hegel MT, Kerns RD (1991) Development of a sleep diary for chronic pain patients. J Pain SymptomManage 6: 65–72. 10.1016/0885-3924(91)90520-e 2007794

[48] Bolger N, Davis A, Rafaeli E (2003) Diary Methods: Capturing Life as it is Lived. Annual Review of Psychology 54: 579–616. 10.1146/annurev.psych.54.101601.145030 12499517

[49] Aaron LA, Turner JA, Mancl L, Brister H, Sawchuk CN (2005) Electronic diary assessment of pain-related variables: Is reactivity a problem? The Journal of Pain 6: 107–115. 15694877 10.1016/j.jpain.2004.11.003

[50] Peters ML, Sorbi MJ, Kruise DA, Kerssens JJ, Verhaak PFM, et al (2000) Electronic diary assessment of pain, disability and psychological adaptation in patients differing in duration of pain. Pain 84: 181–192. 10666523 10.1016/s0304-3959(99)00206-7

[51] Åkerstedt T, Hume K, Minors D, Waterhouse J (1994) The subjective meaning of good sleep, an intraindividual approach using the Karolinska Sleep Diary. Perceptual and motor skills 79: 287–296. 7991323 10.2466/pms.1994.79.1.287

[52] Keklund G, Åkerstedt T (1997) Objective components of individual differences in subjective sleep quality. Journal of sleep research 6: 217–220. 9493520 10.1111/j.1365-2869.1997.00217.x

[53] Harvey AG, Stinson K, Whitaker KL, Moskovitz D, Virk H (2008) The subjective meaning of sleep quality: a comparison of individuals with and without insomnia. Sleep 31: 383. 18363315 10.1093/sleep/31.3.383 PMC2276747

[54] Affleck G, Tennen H, Urrows S, Higgins P (1991) Individual differences in the day-to-day experience of chronic pain: a prospective daily study of rheumatoid arthritis patients. Health Psychology 10: 419–426. 1765037 10.1037//0278-6133.10.6.419

[55] Affleck G, Tennen H, Urrows S, Higgins P (1994) Person and contextual features of daily stress reactivity: individual differences in relations of undesirable daily events with mood disturbance and chronic pain intensity. J PersSocPsychol 66: 329–340. 10.1037//0022-3514.66.2.329 8195989

