# Supplementary material for: Better Quality Sleep Promotes Daytime Physical Activity in Patients with Chronic Pain? A Multilevel Analysis of the Within-Person Relationship
Source: PLoS One. 2014 Mar 25;9(3):e92158. doi: 10.1371/journal.pone.0092158 (PMC3965418; doi:10.1371/journal.pone.0092158)
Supplement: Appendix S1 — Details of the analysis method. (DOCX) [file pone.0092158.s001.docx]

**APPENDIX S1**

Multilevel models generalize standard linear regression analyses by allowing additional flexibility in describing the relationship between the predictor and predicted variable. The predictor can be either a fixed or random effect, as in standard linear regression analyses, but additionally the predictor can be fixed or random at either or both the ‘day’ and ‘participant’ levels. As different model structures can produce different results, we compared the predictors across all possible model structures. We investigated four different possible model structures for each predictor, and each model that included a fixed predictor had a corresponding model in which the fixed predictor term was absent (constant model). All models with more than one random term at each level included all covariance terms. In our analyses, 98% of models converged.

Predictors were assessed for significance by comparing the best fit (maximum likelihood) between models. For nested models, like our constant models,
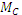

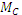
, and constant + predictor models,
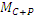

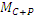
, we can assess the significance of the predictor using a Likelihood Ratio Test,

The significance of a LRT value was found using a χ^2^ distribution with degrees of freedom equal to the difference in the number of parameters between the constant and constant + predictor models.

Unlike the assessment of significance, the assessment of the relative strength of the predictors requires a comparison of non-nested models. Akaike information criterion (AIC; ^56^) gives a standard method for doing so, rewarding goodness of fit but penalising model flexibility. AIC values are calculated using the maximum likelihood fit value and the number of parameters,

where *p(M_i_*) is the likelihood of model *i* and *V_i_* is the number of parameters in the model. AIC gives us an interval scale for comparing models: lower AIC values indicate a better model.

We transformed the raw AIC values to relative model probabilities ^57-59^ to make the differences more intuitive. Relative probability is defined as the ratio of the exponentiated AIC value of a particular predictor over the sum of the exponentiated AIC values across all the of the predictors


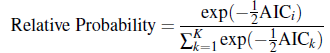


Relative probabilities tell us how likely a model is given the data compared against the other models that we are considering, assuming all models are equally likely a priori.

As a technical aside, instead of using the best model as determined by AIC to find structure-invariant relative probabilities, the relative probabilities for each predictor over the different model structures can be averaged and renormalised. The orderings of predictor strengths were the same between the two approaches.

56. Akaike H. Information theory and an extension of the maximum likelihood principle. In: Petrov BN, Csaki F, eds. Proceedings of the second international symposium on information theory. Budapest: Akademiai Kiado, 1973:267-81.

57. Wagenmakers EJ, Farrell S. AIC model selection using Akaike weights. Psychonomic Bull Rev 2004;11:192-6.

58. Burnham KP, Anderson DR. Model selection and multimodel inference: a practical information-theoretic approach. New York: Springer-Verlag, 2002.

59. Akaike H. On the likelihood of a time series model. The Statistician 1978;27:217-35.
